# Bone gain following loading is site-specifically enhanced by prior and concurrent disuse in aged male mice

**DOI:** 10.1016/j.bone.2020.115255

**Published:** 2020-04

**Authors:** Gabriel L. Galea, Peter J. Delisser, Lee Meakin, Joanna S. Price, Sara H. Windahl

**Affiliations:** a Developmental Biology and Cancer, UCL GOS Institute of Child Health, London, UK; b Comparative Biomedical Sciences, Royal Veterinary College, London, UK; c School of Veterinary Sciences, University of Bristol, Bristol, United Kingdom; d Veterinary Specialist Services, Brisbane, Australia; e Royal Agricultural University Cirencester, Cirencester, United Kingdom; f Department of Laboratory Medicine, Karolinska Institutet, Huddinge, Sweden

**Keywords:** Mechanical loading, Mouse, Bone, Site-specific

## Abstract

The primary aim of osteoanabolic therapies is to strategically increase bone mass in skeletal regions likely to experience high strains. In the young healthy skeleton, this is primarily achieved by bone's adaptation to loading. This adaptation appears to fail with age, resulting in osteoporosis and fractures. We previously demonstrated that prior and concurrent disuse enhances bone gain following loading in old female mice. Here, we applied site specificity micro-computed tomography analysis to map regional differences in bone anabolic responses to axial loading of the tibia between young (19-week-old) and aged (19-month-old), male and female mice. Loading increased bone mass specifically in the proximal tibia in both sexes and ages. Young female mice gained more cortical bone than young males in specific regions of the tibia. However, these site-specific sex differences were lost with age such that bone gain following loading was not significantly different between old males and females. To test whether disuse enhances functional adaption in old male mice as it does in females, old males were subjected to sciatic neurectomy or sham surgery, and loading was initiated four days after surgery. Disuse augmented tibial cortical bone gain in response to loading in old males, but only in regions which were load-responsive in the young. Prior and concurrent disuse also increased loading-induced trabecular thickening in the proximal tibia of old males. Understanding how diminished background loading rejuvenates the osteogenic loading response in the old may improve osteogenic exercise regimes and lead to novel osteoanabolic therapies.

## Highlights

• Sex differences in cortical tissue area of young and old mice are not site-specific.
• The loading response in young, but not old, mice is sex- and site-specific.
• The cortical loading response is site-specifically enhanced by disuse in old mice of both sexes.
• The trabecular loading response can be rescued by disuse in old male, but not female, mice.

## 1 Introduction

Sexual dimorphism in skeletal mass and architecture are well established in mice, humans, and other species [[1], [2], [3], [4], [5], [6], [7], [8]]. Male long bones are generally wider than female bones and trabecular BMD in male mice is higher in the vertebrae, but lower in the tibia compared to female mice [2]. In the young, healthy skeleton, bone adapts to increases in loading-engendered mechanical strains by increasing, mainly periosteal, bone formation. Thereby, the bone mass and architecture are matched to the new level of loading. These homeostatic processes are commonly referred to as the mechanostat [[9], [10], [11]].

Bone loss during ageing is also different between the genders [5,7]. During ageing, both men and women lose cortical bone endosteally, but because men also add more bone periosteally, the total bone loss is higher in women than in men [7]. The loading-induced increase in bone formation is greatly diminished in old mice [[12], [13], [14]], supporting the hypothesis that the ageing-related loss of bone mass reflects a failure of bone to match its structure to its load-bearing function in the old [11,15].

Loading-related bone gain is also diminished in various genetically modified mouse models. For example, deletion of the estrogen receptor α (ERα) reduces loading-related cortical bone gain in adult female mice, but its expression is dispensable in males [16,17]. This and other models suggest that the mechanisms underlying the mechanostat are sex specific. Ageing-related deficiencies in cellular responses to mechanical strain are also sex-specific. Cortical long bone osteoblasts from old males and females proliferate less than their young counterparts following exposure to mechanical strain in vitro, but the cause of this effect is different. Osteoblasts from old male mice fail to enter the cell-cycle in response to strain, whereas female osteoblasts enter the cell-cycle but subsequently arrest in the G2-phase [12,14].

Sex-specificity raises the concern that mechanostat-dependent osteoanabolic therapies intended for the treatment of osteoporosis in women may be ineffective in men. Currently available anabolic treatments for osteoporosis, such as parathyroid hormone and anti-sclerostin antibodies, have been reported to enhance bone gain following loading in female mice [[18], [19], [20]]. We [15] and others [21] have also observed that reducing habitual loading through prior and concurrent disuse “rejuvenates” bone formation old female mice in response to loading in a site-specific manner. Responses were primarily in the proximal tibia of old female mice. Disuse-dependent enhancement of bone gain following loading is compartment specific; it occurs in the proximal tibial cortical bone through periosteal expansion, but not in the trabecular compartment in female mice [15]. The tibia does not expand uniformly along the length of the bone following axial loading. While the proximal tibia expands, the distal tibia does not gain bone following loading in either young or old female mice [22,23].

Proximal versus distal site-specificity in bones' adaptation to loading is now readily quantifiable through application of software tools which quantify cross-sectional bone mass parameters at multiple sites. The previously reported and validated, open source, site-specificity analysis (SSA) tool quantifies standard measures of bone mass and architecture at each 1% site along the bone's length [22]. We have previously applied SSA to discriminate between site-specific and global changes in cortical mass in response to changes in loading, ageing, genetic mutations and hormonal treatment [19,22,24]. Here, we applied SSA to map baseline and loading-induced, sex- and site-specific differences in the mouse tibial cortex. In addition, we used SSA to determine whether disuse improves the bone response to loading in old males as it does in female mice.

## 2 Material and methods

### 2.1 Animals

Young (19-week-old) and old (19-month-old) female and male C57BL/6J mice were obtained from Charles River Inc. (Margate, UK) and housed in a standard animal facility under controlled temperature (22 °C) and photoperiod (12 h of light, 12 h of dark) and fed pellet diet containing 0.75% calcium (EURodent Diet 22%; PMI Nutrition International, LLC, Brentwood, MO, USA) ad libitum. All procedures complied with the UK Animals (Scientific Procedures) Act 1986 under a UK Government Home Office project license (PPL30/2829) and were reviewed and approved by the University of Bristol ethics committee (Bristol, UK).

### 2.2 Ex vivo strain measurements

**Supplemental Fig. S1:**
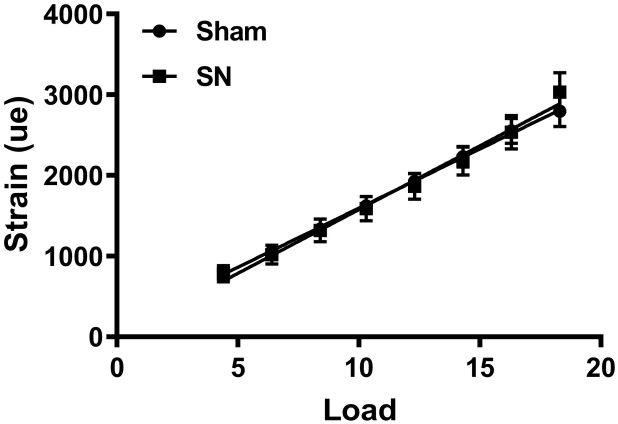
Four days unloading does not affect bone strength. Strain:load curves of tibiae 4 days post sham or neurectomy (SN) surgery of old male mice.

The magnitude of axial mechanical strain applied to the tibia during loading was established ex vivo to facilitate application of similar magnitudes of peak strain to all groups of mice. Sciatic neurectomy (SN, N = 6) or sham (N = 6) surgery was performed on the right limb as previously described [25,26]. The mice were killed four days post-surgery and directly used for the ex vivo strain measurements as previously described [27,28]. Strains were measured across a range of peak compressive loads between 4 and 18 N (Supplemental Fig. S1). These peak loads were applied with the same ramped trapezoidal waveform, using the same 3100 ElectroForce® Test Instrument (Bose Corporation, MN, USA) with the same holding cups that were used later for in vivo loading. From the data, a linear regression analysis was performed.

### 2.3 Mechanical loading

**Fig. 1:**
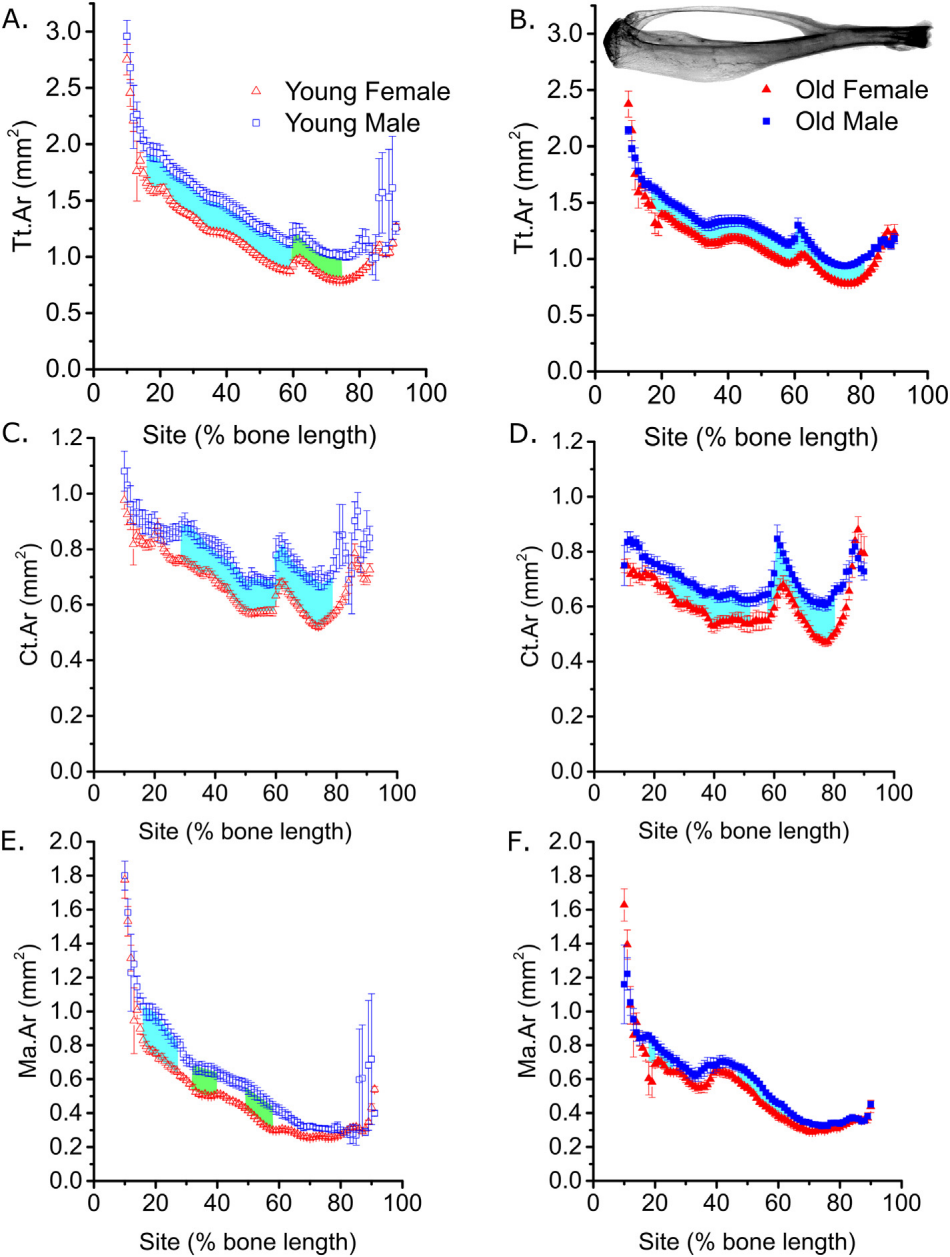
The sex differences in cortical tissue area of young and old mice are not site-specific. Site-specificity analysis of (A and B) periosteally enclosed area (Tt.Ar), (C and D) cortical area (Ct.Ar), (E and F) marrow area (Ma.Ar), of young (A, C and E) and old (B, D and F) male and female mice. Points represent the mean ± SEM. Shaded regions indicate regions of statistical difference between the sexes. Turquoise shading p < 0.05, green shading p < 0.10. N = 6–7 and N = 12–13 for young and old mice respectively. (For interpretation of the references to colour in this figure legend, the reader is referred to the web version of this article.)

**Fig. 2:**
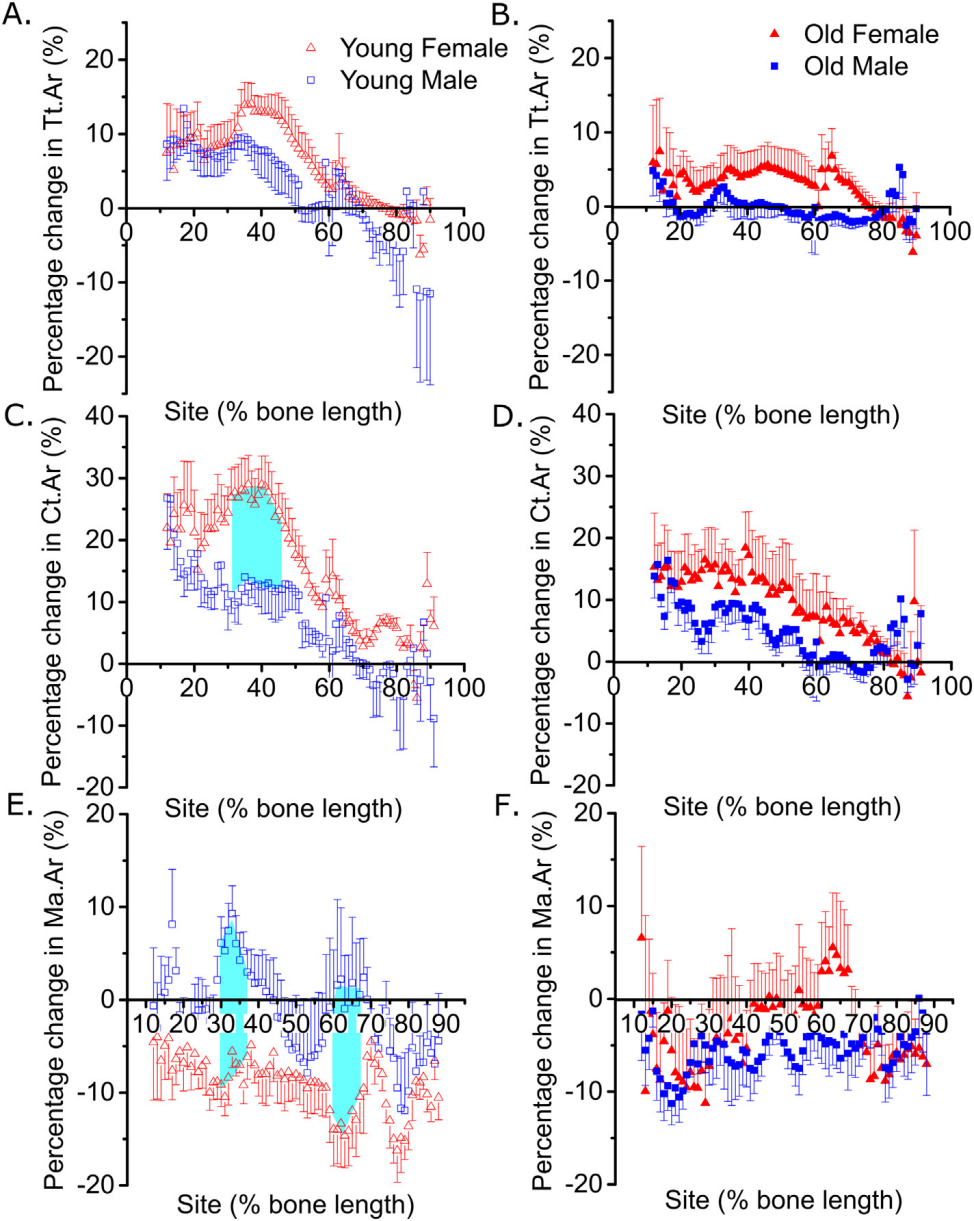
The loading response in young, but not old, mice is sex- and site-specific. Site-specificity analysis of the loading-related percent change in (A and B) periosteally enclosed area (Tt.Ar), (C and D) cortical area (Ct.Ar), (E and F) marrow area (Ma.Ar) of young male and female (A, C and E), and old male and female (B, D and F) mice. Points represent the mean ± SEM of the relative difference in the loaded right side vs. the non-loaded left side control. Turquoise shaded regions indicate regions of statistical difference between the sexes where p < 0.05. N = 6–7 and N = 12–13 for young and old mice respectively.

**Fig. 3:**
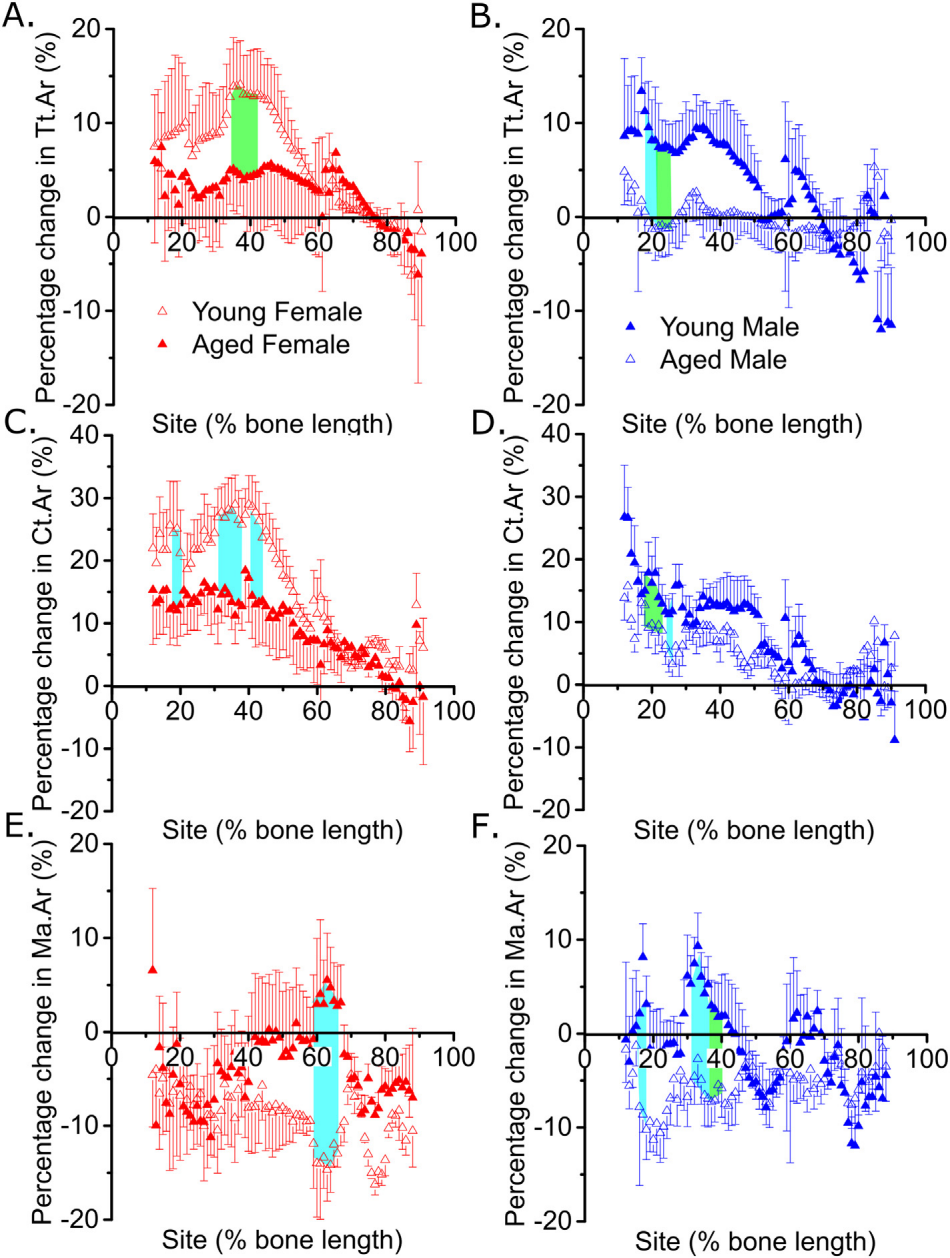
The decreased loading response in old mice is sex- and site-specific. Site-specificity analysis of the loading-related percent change in (A and B) periosteally enclosed area (Tt.Ar), (C and D) cortical area (Ct.Ar), (E and F) marrow area (Ma.Ar) of young and old female (A, C and E) and young and old male (B, D and F) mice. Points represent the mean ± SEM of the relative difference in the loaded right side vs. the non-loaded left side control. Shaded regions indicate regions of statistical difference. Turquoise shading p < 0.05, green shading p < 0.10. N = 6–7 and N = 12–13 for young and old mice respectively. (For interpretation of the references to colour in this figure legend, the reader is referred to the online version of this chapter.)

The protocol for non-invasively loading the mouse tibia has been reported previously [[27], [28], [29], [30]]. In short, right tibias were subjected to external mechanical loading under isoflurane-induced anesthesia on alternate days for 2 weeks and mice were sacrificed 3 days following the last period of loading. In order to compare bone's responses to loading between sexes and ages, young and old male and female C57BL/6J mice (n = 6 per group) were subjected to loading so as to engender a peak strain of 2500 με on the medial tibial surface as reported in [14]. Site specificity analyses of the tibiae from these mice are reported in Fig. 1, Fig. 2, Fig. 3.

For neurectomy experiments in old male mice, loading started 4 days post-surgery. All mice were allowed normal cage activity between loading sessions. The strain:load relationship was not significantly altered four days after the sham or SN surgeries (Supplemental Fig. S1A). Both sham and SN old male mice were therefore loaded with the same peak load during the experiment (14.5 N) to apply 2270 με at the start of the loading experiment.

**Fig. 4:**
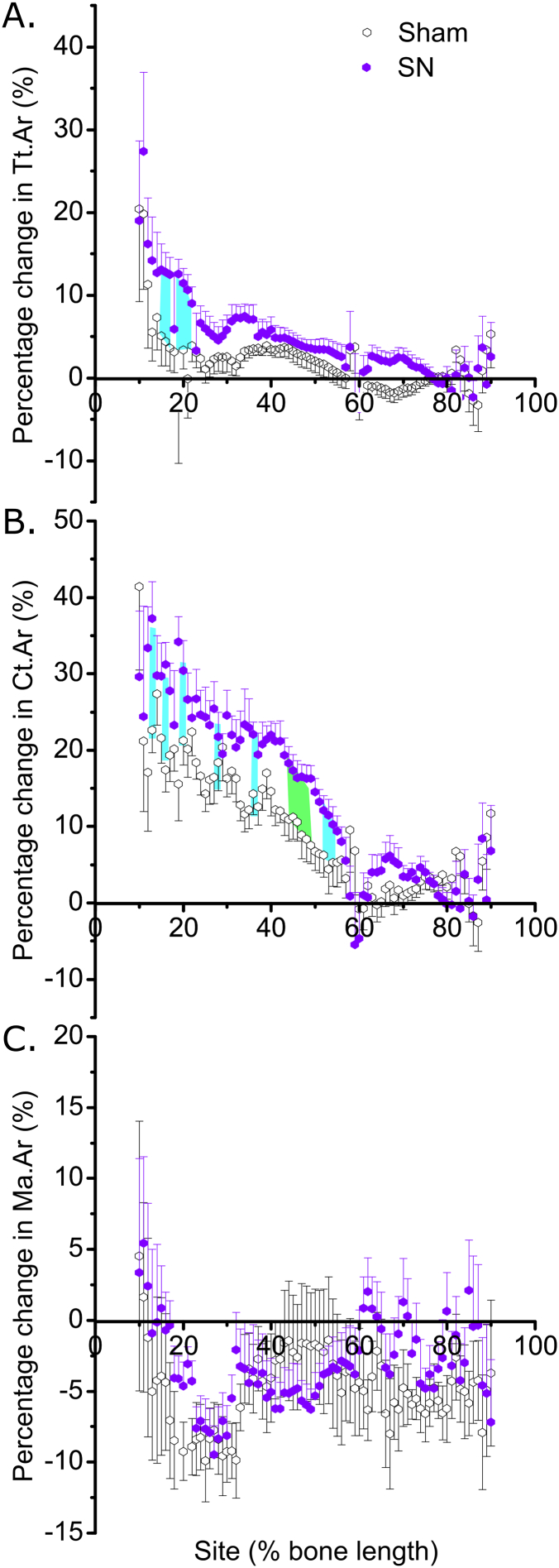
Concurrent disuse rescues the age-dependent reduced loading response in male mice. Site-specificity analysis of the loading-related percent change in (A) periosteally enclosed area (Tt.Ar), (B) cortical area (Ct.Ar), (C) marrow area (Ma.Ar) of sham and SN old male mice. Points represent the mean ± SEM of the relative difference in the loaded right side vs. the non-loaded left side control. Shaded regions indicate regions of statistical difference. Turquoise shading p < 0.05, green shading p < 0.10. N = 13 for sham and N = 12 for SN. (For interpretation of the references to colour in this figure legend, the reader is referred to the web version of this article.)

**Fig. 5:**
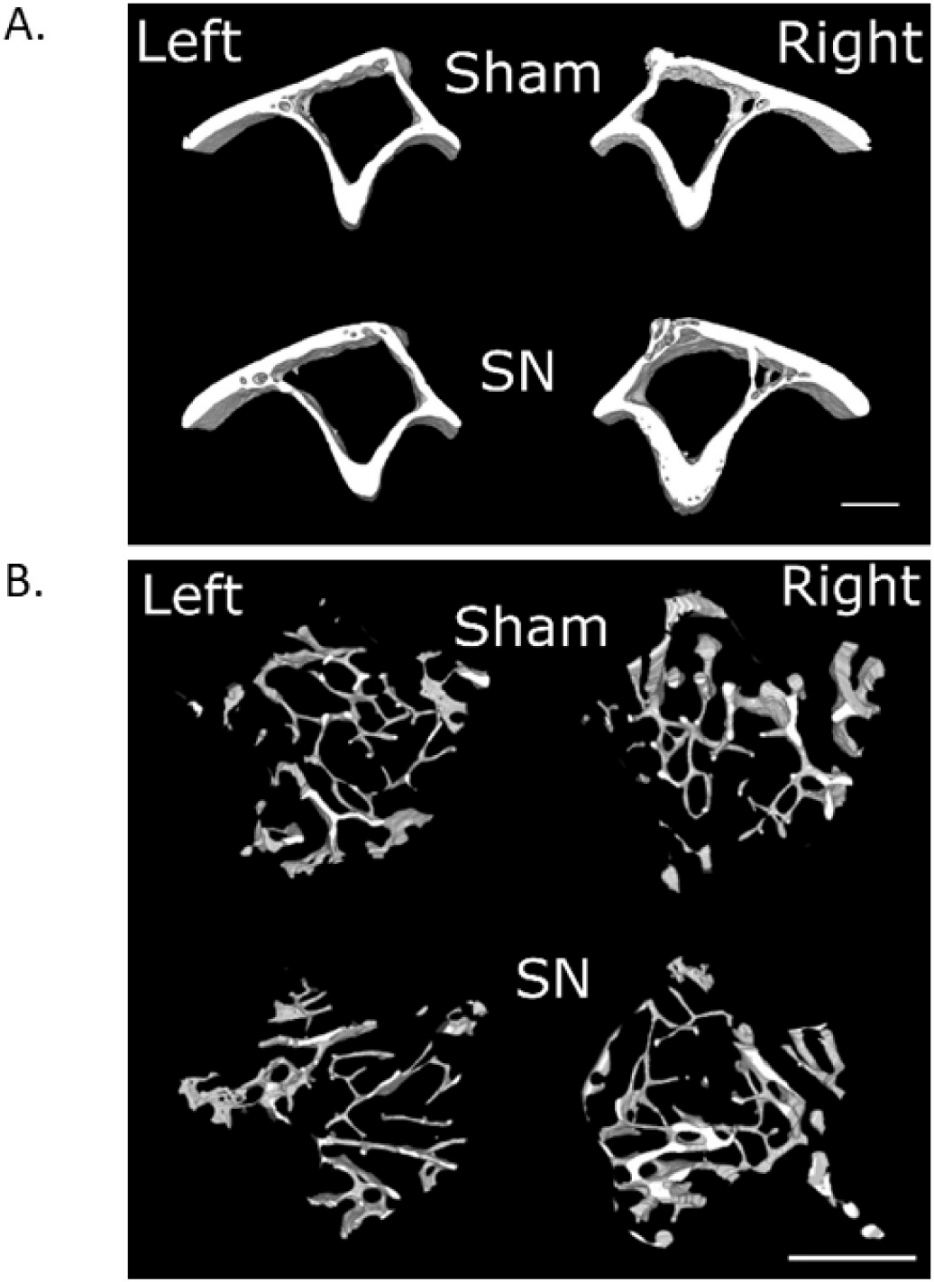
Representative μCT images. Representative images of (A) cortical bone at the 37% region and (B) trabecular bone in the proximal metaphyseal region of tibia. Right legs were subjected to axial loading, while the left legs are internal controls. SN = sciatic neurectomy. Scale bar = 1 mm.

**Fig. 6:**
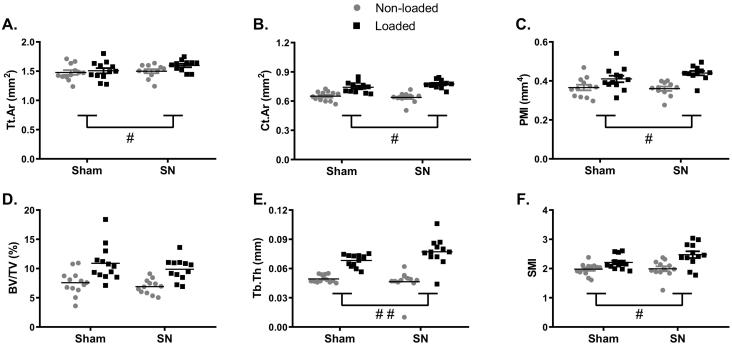
Conventional μCT confirms that concurrent disuse rescues the age-dependent reduced loading response in old male mice. Conventional μCT analysis of (A) periosteally enclosed (Tt.Ar), (B) cortical area (Ct.Ar), (C) polar moment of inertia (PMI), (D) trabecular bone volume (BV/TV), (E) trabecular thickness (Tb.Th) and (F) structure model index (SMI) are shown for sham and neurectomized (SN) mice. Left non-loaded tibiae are represented by white boxes and right loaded tibiae are indicated by black boxes. N = 13 for sham and N = 12 for SN. *p < 0.05, **p < 0.01, ***p < 0.001 comparing right and paired left limbs. #p < 0.05, ##p < 0.01 comparing the percentage change due to loading between Sham and SN mice.

To determine whether disuse alters the response to loading in old males as it does in females [15], 19-month-old C57BL/6J male mice were divided in two weight-matched groups. The first group (N = 13) was subjected to bilateral sham surgery and unilateral loading of the right tibia to demonstrate the effect of loading on a background of normal locomotion. The second group (N = 12) was subjected to unilateral sham (left leg) and unilateral SN (right leg) surgery followed by loading of the right tibia to demonstrate the effect of loading on a background of disuse. All mice were kept in separate cages from the day of surgery so that fighting could not affect the loading response [31]. Four days post-surgery, the right tibias were subjected to external axial mechanical loading, every second day (in total 8 periods of loading), under isoflurane-induced anesthesia, to induce bone formation. Left limbs were used as internal controls as previously validated [29,30]. An SN-only control group was deemed unnecessary given SN is well established to cause loss of bone mass only in the disused limb [14,15,26,32]. Effective SN was determined by measurement of wet gastrocnemius muscle mass in each mouse. The mice were killed on the third day after the last loading, and the tibiae were dissected and fixed in 4% PFA for 48 h. The tibiae were then stored in 70% EtOH until analysis by micro computed tomography (μCT). Analyses of this aged male “rescue” experiment are shown in Fig. 4, Fig. 5, Fig. 6.

### 2.4 High resolution μCT measurements of standard bone measurements

Whole tibiae were imaged using the SkyScan 1172 (Bruker, Kontich, Belgium) with a voxel size of 4.8 μm (110 μm^3^). The scanning, reconstruction and method of analysis has been previously reported [31,33].

### 2.5 Site specificity analysis

SSA was performed and analyzed as previously described [22] and statistically analyzed using linear mixed models in SPSS (IBM, v.22). Bone site was used as a fixed categorical parameter, the intervention (loading, surgery) as a fixed effect, and an intervention by site interaction to account for site-specific responses. Mouse ID was included as a random effect with repeated measures at different sites from each mouse accounted for in the model. When the effect of the intervention was significant overall, a post-hoc Bonferroni correction was applied to identify individual sites where differences reached statistical significance. p < 0.05 was considered significant, but when several contiguous sites each tended towards significance (p < 0.1), these are also indicated.

### 2.6 Statistical analysis

Data is presented as mean ± SEM or as individual data points. Except when using SSA (statistical analysis described above), to evaluate the effect of loading (right loaded vs. left non-loaded control), surgery (sham vs. SN) and their interaction “loading*surgery”, a repeated-measures ANOVA was used with a post-hoc, paired *t*-test within groups. The effects of body weight, gastrocnemius muscle wet weight and % changes to loading were also evaluated by paired *t*-tests (left versus right within each mouse). An effect was considered significant at p < 0.05.

## 3 Results

### 3.1 Sex differences in cortical tissue area of young and old mice are minimally site-specific

We first compared cortical bone of young and old, male and female mice using SSA. In both young and old mice, total tissue area (Tt.Ar) was greater in male than female mice in a non-site specific manner (sex by site interaction p = 0.991 and p = 0.61, Fig. 1A and B respectively). Similarly, cortical area (Ct.Ar) was significantly larger in male mice compared to female mice along most of the length of the tibia independently of age (Fig. 1C and D). However, the magnitude of this difference was site specific in the young (interaction p = 0.023), but not old (p = 0.52) mice.

Also independently of age, marrow area (Ma.Ar) was larger in tibiae from males compared with females primarily in the proximal diaphysis, but not detectably so in the distal tibia, where the marrow cavity is small (Fig. 1E and F). The difference in Ma.Ar was not significantly site-specific in the young (interaction p = 0.470), but was significantly site-specific in the old (p = 0.04).

### 3.2 The loading response in young, but not old, mice is sex- and site-specific

In young male and female mice, Tt.Ar increased following loading primarily in the proximal tibia (site by loading interaction p < 0.001 for both sexes). There was no significant difference in the loading response in Tt.Ar in male compared to female mice (overall p = 0.75 comparing male vs. female), although female mice tended to show a greater percentage increase on average around 37% of the bone's length from the proximal end (Fig. 2A). The loading-related increase in Ct.Ar was also site-specific in both sexes (site by loading interaction p < 0.01), and was significantly greater in the proximal to central diaphysis of female compared to male tibiae (Fig. 2C). In females, maximum responsiveness was observed around the 37% site, as we have previous described [22]. However, in young male mice, no such “peak” was observed (Fig. 2C). Loading was also associated with a greater reduction in Ma.Ar in female than male mice at 30–36% and 60–68% of the bone's length from the proximal end (Fig. 2E). Taken together, these findings demonstrate that the adaptive cortical responses following axial loading are site-specific in both sexes but are more marked and follow a different spatial pattern in female than male mice subjected to the same peak strain stimulus. In old mice, the loading-related change in Tt.Ar (Fig. 2B), Ct.Ar (Fig. 2D) and Ma.Ar (Fig. 2F) was not significantly different overall between males and females.

### 3.3 The decreased loading response in old mice is sex- and site-specific

In old mice, the overall loading-related increase in Tt.Ar and Ct.Ar was confirmed to be smaller than in young mice of either sex, as we have previously reported [14]. However, SSA provided substantial additional information. The loading-related increase in Tt.Ar and Ct.Ar tended to be diminished in the old versus young females specifically around the peak responsiveness site, approximately 37% of the bone's length from the proximal end (Fig. 3A and B). In males, a significant age-related difference in the loading-related change in Tt.Ar and Ct.Ar was detected more proximally, at around 20% of the bone's length (Fig. 3D and E) and not the commonly-analyzed 37% or 50% sites.

Ma.Ar decreased following loading in both young and old female mice in the proximal tibia, but the reduction was more marked distally in the young mice (Fig. 3C), as we have previously reported [22]. This distal reduction in Ma.Ar was not observed in old female mice (Fig. 3C). Old male mice showed significantly greater reductions in Ma.Ar in the proximal tibia than young mice (Fig. 3F).

### 3.4 Disuse site-specifically enhances the cortical adaptive responses to loading in old males

**Supplemental Fig. 2:**
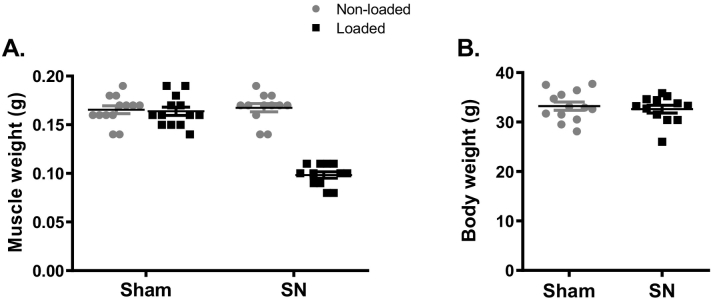
Muscle weight, but not body weight is affected by neurectomy. (A) Gastrocnemius and (B) body weights are shown for sham operated and neurectomized (SN) mice. Only the right loaded limbs in the SN group were neurectomized. ***p < 0.001 for non-loaded vs. loaded tibiae of the same treatment.

SN significantly reduced gastrocnemius muscle wet weight in the right leg (−41%, p < 0.001) compared to the left control leg of SN mice (Supplemental Fig. 2A), confirming that the surgery was successful. The overall body weight was not affected by SN (Supplementary Fig. 2B).

We have previously shown that SN-induced prior and concurrent disuse can “rescue” the age-related decline in cortical bones' response to loading in female mice. To establish if this response is sex-specific, we used SSA to investigate the effect of prior and concurrent disuse on the loading response in old male mice. Loading on a background of prior and concurrent disuse resulted in a significantly greater increase in Tt.Ar predominantly around the 20% site in old males (Figs. 4A and 6A), the region which showed the greatest age-related decline in responsiveness. Similarly, the increase in Ct.Ar was significantly greater in SN- than sham-operated mice in the proximal tibia, including significant enhancement at the 20% site (Figs. 4B and 6B). In contrast, the reduction in Ma.Ar observed at ~20–30% of the bone's length in old male mice was not significantly altered by disuse (Fig. 4C).

These findings were validated by conventional μCT analysis selectively at the cortical 20% site (Figs. 5A and 6, and Table 1). Other standard measures of cortical bone mass were also assessed. Cortical cross-sectional thickness (Cs.Th, Table 1) and cortical area/periosteally enclosed area (Ct.Ar/Tt.Ar, Table 1) increased following loading irrespective of SN, whereas polar moment of inertia (PMI, Fig. 6C) increased to a greater extent in the SN versus Sham group relative to the left limbs. Thus, prior and concurrent disuse enhances the cortical osteogenic response to loading in old male mice as we have previously reported in old females [15]. This “rescue” is site-specific and most pronounced at the cortical 20% site in males, where it was readily validated by conventional μCT analysis (Figs. 5A and 6A–C, and Table 1).

**Table 1:** Cortical and trabecular parameters measured using conventional μCT.

	Sham		Sciatic neurectomy	
	Non-loaded	Loaded	Non-loaded	Loaded
B.Ar/Tt.Ar	2.253 ± 0.065	2.030 ± 0.045***	2.345 ± 0.031	2.072 ± 0.035***
Ma.Ar	0.817 ± 0.035	0.766 ± 0.034*	0.857 ± 0.021	0.825 ± 0.024
Cs.Th	0.108 ± 0.003	0.120 ± 0.005*	0.100 ± 0.006	0.122 ± 0.003**
Ct.Po	2.262 ± 0.187	2.602 ± 0.635	3.382 ± 1.679	2.999 ± 0.718
Tb.N	1.535 ± 0.117	1.587 ± 0.104	1.751 ± 0.326	1.308 ± 0.079
Tb.Sp	0.293 ± 0.011	0.286 ± 0.009	0.266 ± 0.024	0.302 ± 0.008
Tb.Pf	23.04 ± 0.87	20.00 ± 0.95**	23.80 ± 0.90	20.68 ± 1.02

Conventional μCT analysis of periosteally enclosed bone area (B.Ar/Tt.Ar), marrow area (Ma.Ar), cortical cross-sectional thickness (Cs.Th), cortical porosity (Ct.Po), trabecular number (Tb.N), trabecular spacing (Tb.Sp) and trabecular patterning factor (Tb.Pf). N = 13 for sham and N = 12 for SN. *p < 0.05, **p < 0.01, ***p < 0.001 comparing right and paired left limbs.

### 3.5 Disuse enhances the trabecular adaptive responses to loading in old male mice

We also investigated the effect of prior and concurrent disuse on the loading-induced trabecular bone gain. Loading significantly increased trabecular bone volume fraction (BV/TV), trabecular thickness (Tb.Th) and structure model index (SMI, Figs. 5B and 6D–F), but decreased the trabecular patterning factor (Tb.Pf, Table 1) in both sham and SN tibiae of old male mice. Trabecular number (Tb.N) and trabecular separation (Tb.Sp) did not change in either group (Table 1). Prior and concurrent disuse significantly augmented the loading-induced increase in Tb.Th and SMI (Fig. 6E and F).

## 4 Discussion

While skeletal sexual dimorphism is well established, sex-specific mechanisms underlying the bone anabolic effects of loading are only now being explored. These sex-specific mechanisms are masked or exaggerated when bone mass is only assessed at a few cross-sectional sites. Most previous studies into bone's adaptation to loading selected the sites to analyze based on convenience (e.g. mid-diaphysis [[34], [35], [36]]) or maximal responses observed in female mice (i.e. tibial 37% site [19,28,37]). Pre-selection of sites for analysis is particularly prominent in the era of in vivo μCT scanning, which is currently limited to assessing a narrow cross-section of bone. Ex vivo μCT analysis remains invaluable as it enables quantification of bone mass along the bone's entire length and remains the method of choice to characterize genetic mutations or ageing phenotypes.

Whole-tibia cortical analyses have shown that “systemic” interventions such as PTH treatment [19] and genetic mutations [24,38,39] influence bone mass site-specifically. In contrast, ovariectomy diminishes cortical area uniformly along the length of the tibia [22]. Here we show that young male tibiae have uniformly larger tissue area than those in female mice. Intriguingly, cortical area is minimally dimorphic at the extreme ends of the bone, but significantly larger in males along the rest of the diaphysis. This difference may be due to differences in strain damping associated with tibial curvature [40], differential load bearing between cortical and trabecular compartments, or regional responses to sex hormones. The magnitude of these sex-related differences are less marked in the aged mice, whose bone mass is lower in both sexes [41].

In young mice, loading increases cortical bone mass in site- and sex-specific patterns. Previous studies reported a larger load-related increase in cortical bone mass in young female mice compared to young male mice at the 37% site [14,17]. We further confirm these findings here, showing a distinct peak responsiveness at 37% of the bone's length in female mice which is not evident in males. Loss of medullary area, indicative of enhanced endosteal bone formation, was also significantly greater in young females than males proximally and mid-distally. However, it is not simply the case that male mice are generally less responsive to loading than female mice; loading-related changes in cortical parameters were comparable between young males and females at the proximal end of the tibia.

Sex-specific hormone levels decrease with ageing [42]. Concurrently, the sex-specific response to loading is decreased in old mice. This raises the hypothesis that sex hormones direct site-specific functional adaptation to loading. We and others have shown that estrogen receptor α (ERα) is essential, while endogenous estrogens are not important, for the cortical osteogenic effects of loading in female rodents [16,17,28,[43], [44], [45]]. In addition, serum estradiol levels are not decreased in female mice during ageing [42]. Taken together, this indicates that the reduced loading response in old female mice is not due to decreased estrogen levels. The osteogenic effects of loading is enhanced when the androgen receptor (AR) is disrupted, or testosterone is removed by orchidectomy, in young adult male mice [16,46]. In line with these results, testosterone and dihydrotestosterone reduce the osteogenic effects of loading [46]. Together, this indicates that androgens inhibit the osteogenic effects of loading through AR. Surprisingly, both testosterone and dihydrotestosterone are drastically decreased in male mice during ageing [42]. This indicates that the altered androgen levels are not likely to cause the reduced loading response in old male mice. Bone mass is also influenced by other hormones linked to sexual determination or reproductive function, such as progesterone [47] and follicle stimulating hormone [48]. It is largely unknown how these hormones interact with the loading response in young and old males or females.

In young females, previous loading studies reported either a decrease [34] or no change [30] in medullary area following loading when analyzing pre-selected sites. Site-specificity analysis may reconcile these discrepancies as loading reduces medullary area significantly more immediately distal to the tibia/fibula junction (~60%) in young compared to old female mice. Although, young males showed minimal changes in medullary area at any site in response to loading, the loading response was significantly greater in old males primarily in the proximal tibia where the marrow area was reduced in response to load. While unexpected, this apparent age-related enhancement of medullary responsiveness to loading specifically in males is in line with our previous findings [14]. Thus, ageing diminishes both periosteal (Tt.Ar) and endosteal (Ma.Ar) expansion in females whereas it diminishes periosteal but enhances endosteal expansion in males. This is consistent with previous findings that impaired periosteal new bone formation is the primary ageing-related deficiency which limits the mechanostat [13,14].

Our previous findings, that prior and concurrent disuse rejuvenates the mechanostat in cortical bone of old female mice, have now also been independently replicated by others [21]. Here, we demonstrate that this rejuvenation extends to old male mice as well. Disuse enhanced the loading-induced increase in total tissue area and overall cortical area in the proximal tibia of old mice. Additionally, this rejuvenation may be sexually dimorphic in the trabecular compartment. Disuse does not augment trabecular bone gain following loading in old female mice [15]; however, disuse augmented trabecular thickness in the proximal tibia of old male mice. The mechanisms by which disuse alters the osteogenic context in old mice are unknown. As in our previous studies [26], neurectomy-induced muscle catabolism occurred concomitantly with loading-induced bone gain. Although we cannot exclude the possibility that muscle catabolism releases growth factors, such as latent TGF-β, which stimulate bone formation in old mice, we suggest that the main mechanisms whereby disuse alters the loading response in old mice are related to activation of periosteal and endosteal osteoblast function. In young mice, loading induces bone formation independently of resorption [33]. In addition, we have previously reported that disuse-induced rescue of bone's response to loading in old female mice is due to increased periosteal and endosteal mineral apposition rate [15], implicating osteoblasts rather than osteoclasts as effectors of the increased bone mass in response to loading. It is now clear that mechanoresponsive cells in bone of old mice, primarily osteocytes and osteoblasts, remain able to sense mechanical stimuli and rapidly initiate transcriptional responses [14,49]. In addition, osteocytes in the bones of old mice convert loading-related stimuli into appropriate downregulation of the anti-osteogenic Wnt antagonist sclerostin [14]. Thus, prior and concurrent disuse is unlikely to rejuvenate the osteogenic effects of loading by changing bone cells' ability to sense mechanical stimuli, but rather by redirecting cells' ability to transduce those mechanical stimuli into functionally relevant new bone formation.

Despite the current lack of a clear mechanism, the reproducible phenomenon of disuse-induced rejuvenation of the mechanostat in mice raises clinically relevant questions. For example, does non-osteogenic habitual activity blunt bone gain in response to novel strain distributions in the elderly? Does advising patients to increase their physical activity change their response to treatments such as anti-sclerostin antibodies which are mechanostat-manipulating? Exercise interventions are now established as safe and effective in maintaining bone mass [50], but our findings suggest that baseline loading levels should be considered as potential confounding factors in patients' responses to exercise. In conclusion, this study suggests that brief bouts of intense physical activity may increase bone mass in otherwise sedentary elderly men and women.

The following are the supplementary data related to this article.
